# Controlled Size Oils Based Green Fabrication of Silver Nanoparticles for Photocatalytic and Antimicrobial Application

**DOI:** 10.3390/antibiotics12071090

**Published:** 2023-06-22

**Authors:** Seemab Pervaiz, Iram Bibi, Wajid Rehman, Hadil Faris Alotaibi, Ahmad J. Obaidullah, Liaqat Rasheed, Mohammed M. Alanazi

**Affiliations:** 1 Department of Chemistry, Hazara University, Mansehra 21120, Pakistan; seemab.zeb@gmail.com (S.P.); iram_khan1985@yahoo.com (I.B.); liaqatrasheed5142@gmail.com (L.R.); 2 Department of Conservation Studies, Hazara University, Mansehra 21120, Pakistan; 3 Department of Pharmaceutical Sciences, College of Pharmacy, Princess Nourah bint Abdulrahman University, Riyadh 11671, Saudi Arabia; hfalotaibi@pnu.edu.sa; 4 Department of Pharmaceutical Chemistry, College of Pharmacy, King Saud University, Riyadh 11451, Saudi Arabia; aobaidullah@ksu.edu.sa (A.J.O.); mmalanazi@ksu.edu.sa (M.M.A.)

**Keywords:** silver nanoparticles (Ag-NPs), clove oil, cinnamon oil, cardamom oil, agglomeration, size homogeneity and crystallite size

## Abstract

The particle size at the nanometric level allows the manifestation of remarkable properties, chiefly due to changes in surface-to-volume ratio. This study is attributed to the novel green synthesis of nano silver by using essential oils as a capping and reducing agent. Clove oil, cinnamon oil, and cardamom oil were selected for the eco-friendly and low-cost fabrication of silver nanoparticles. The prepared nanoparticles were characterized by photoluminescence spectroscopy, FT-IR spectroscopy, X-Ray diffraction, energy dispersive X-ray spectroscopy, dynamic laser light scattering, thermogravimetric analysis, and transmission electron microscopy. It was found that samples prepared by using cinnamon oil (20 nm) and cardamom oil (12 nm) had smaller particle sizes as compared to those synthesized by using clove oil (45 nm). All the prepared samples exhibited very strong antimicrobial activities with a clear zone of inhibition (6–24 mm) against *Staphylococcus aureus*, *Klebsiella pneumoniae,* and *Candida albicans.* Very resilient photocatalytic activities of the samples were observed against Allura red and fast green dyes. It was concluded that the cinnamon oil-based system is the best size reducer and size homogenizer (less chances of agglomeration) as compared to clove oil and cardamom oil (more chances of agglomeration) for the synthesis of silver nanoparticles.

## 1. Introduction

Green synthesis basically involves the replacement or elimination of destructive materials during a chemical reaction [1]. Ag nanoparticles are known as reliable materials due to their versatile physical and chemical nature and their applications in various fields such as the electronic industry, medicine, catalysis, food preservation, etc. [2,3,4]. Thus, eco-friendly and safe alternatives are dominating the traditional unfriendly approaches especially the controlled shape and size preparations of silver nanoparticles are very important for their antimicrobial applications [5]. Silver nanoparticles have the unique property of eliminating microorganisms by preventing their cellular respiration and consequently their replication. Due to their high bactericidal properties in combination with low toxicity towards living organisms, this opens the door for novel green synthesis of silver nanoparticles through inexpensive and environmentally friendly processes [6]. Silver nanoparticles have the ability to destroy the intracellular metabolic activity thus resulting in leakage of cellular material and breakage of cellular morphology. Moreover, Ag-NPs also generate reactive oxygen species that react with various proteins and DNA to stop DNA synthesis and metabolism [7,8]. Essential oils used for the preparation of NPs form microemulsions which act as nanoreactors that reduce the polydispersity in the particle size [9]. The literature suggests that the concentration of metallic precursor, reduces the power of the reducing agents, and phase dispersion basically controls the size and its heterogeneity [10]. Silver nanoparticles exhibit various shape- and size-dependent properties which have widespread applications in different fields such as catalysis, computer transistors, fuel cells, biosensors, optics, medical imaging, antimicrobial activity, etc. [11]. Silver nanoparticles synthesized by using the green synthesis method show greater antimicrobial activity as compared to those synthesized by chemical means [12].

In this study, essential oils have been used as an eco-friendly alternative to the harmful and toxic chemical-reducing agents for the synthesis of silver nanoparticles. Clove oil is widely used for its antibacterial, antifungal, antioxidant, anesthetic, and analgesic properties [13]. Clove oil contains a total of 23 constituents out of which eugenol (76.8%) is the major component that is actually responsible for the reduction and stabilization of nanoparticles [14] along with 17.4% (β-caryophyllene), 2.1% (α-humulene), and 1.2% (eugenyl acetate) as the important components [15]. Cinnamon oil is traditionally used as a flavoring agent in desserts, chocolates, liqueurs, tea, and coffee and is also used as medicine to cure colds, and coughs, to treat diarrhea, etc. Thus, cinnamon oil also exhibits both antioxidant [16] and antimicrobial activities [17]. Its chemical composition includes cinnamaldehyde as a major constituent along with eugenol, beta-caryophyllene, etc. [18]. The major components of cardamom essential oil comprise 36.61% α-terpinyl acetate, 30.42% 1,8-cineole, 5.79% linalyl acetate, and 4.85% sabinene [19]. Cardamom oil possesses inhibitory activity against various fungal species, insecticidal activity, antispasmodic activities, etc. [20].

This study was conducted to evaluate the reduction capacity, size polydispersity, and stabilizing activity of these three essential oils during NPs synthesis. NPs size plays a critical role in nanoparticle surface modification, cell nanoparticle binding, intracellular trafficking, and tissue diffusion, and enhances targeted drug delivery [21]. Surface composition and charge basically determine the stability of silver nanoparticles and the size of the nanoparticle influences its interaction with the substrate and contact area [22]. Below 10 nm the activity of Ag-NPs is solely by itself while in the case of large particle sizes the activity is attributed to the silver ions [23]. Reduced particle size displays potent antimicrobial activities by controlling the pH of the solution for the Ag-NPs preparation [24]. So, there is a need to introduce a simple green synthesis method to obtain controlled size and uniformity in the sample. Knowing the antimicrobial activities of these three essential oils in combination with Ag-NPs synthesized, antimicrobial activities were studied against *S. aureus*, *Klebsiella pneumoniae,* and *Candida albicans* as well as photocatalytic activities were carried out by using Allura red and fast green dye.

## 2. Materials and Method

All the chemicals used in this research work were purchased from Sigma Aldrich. The materials used were silver nitrate (AgNO_3_, 99.99%), ether (C_2_H_5_)_2_O, 99.9%), sodium borohydride (NaBH_4_, 98%), and ethanol (C_2_H_5_OH, 99.9%). Clove oil, cinnamon oil, and cardamom oil were purchased from Haq Planters in Abbottabad, Pakistan. They were of high purity and used as received. Allura red and fast green dye were used as purchased from Sigma Aldrich for dye degradation. The whole synthesis was carried out by using deionized water.

### 2.1. Green Synthesis of Silver Nanoparticles

**Figure 1:**
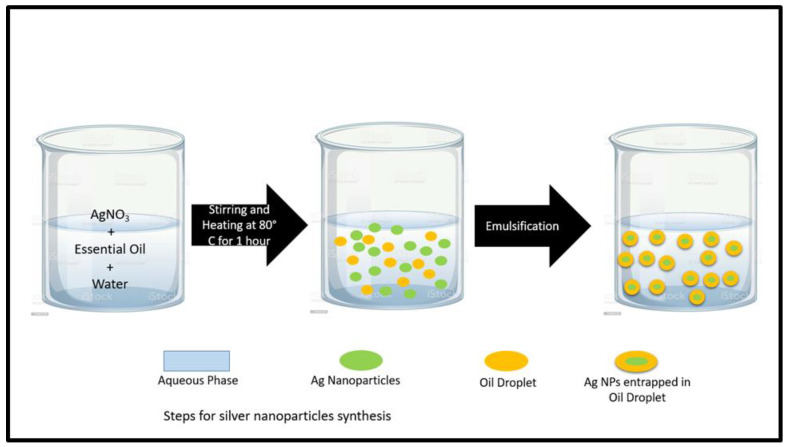
Steps for silver nanoparticles synthesis.

Silver nanoparticles had been synthesized by a simple one-pot reaction. Three samples were prepared and generally named as A1, A2, and A3. Three beakers were utilized: in the first beaker, 1 g of AgNO_3_ was dissolved in 100 mL of deionized water, 2 mL of clove oil was added as a reducing agent, and the beaker was marked as A1; similarly, in the second beaker, the same quantity of silver nitrate was dissolved in water with 2 mL of cinnamon oil as a reducing agent and the beaker was marked as A2; then, in the third beaker, 2 mL of cardamom oil was added to the same quantity of salt and water and it was marked as A3. These three beakers were then continuously stirred and heated at 80 °C for 1 h. After 1 h, the color of the solution present in the beakers was changed from transparent to dark brown. This color change indicates the formation of silver nanoparticles. Finally, the nanoparticles were washed with ether to remove the oils followed by washing with distilled water and then dried in an oven at 80 °C as shown in Figure 1.

### 2.2. Characterization of Silver Nanoparticles

The formation of Ag-NPs was confirmed by using photoluminescence spectroscopy (Perkin Elmer LS55). Readings were taken at a rate of 800 nm per minute while the wavelength ranged from 800 to 200 nm. Allura red and fast green dye degradation was carried out by using a Shimadzu UV-1700 UV-Vis spectrophotometer to examine the absorption spectra and catalytic activities of the samples. The size of NPs and polydispersity index were measured in quartz cuvettes at 90 degrees at room temperature by using dynamic laser light scattering (Brookhaven Ins. and Cor. 90 plus particle size analyzer). The morphology, dimension, and shape of NPs were investigated by using transmission electron microscopy (JEM-2100, JEOL, Japan). To investigate the stability of the nanomaterials at higher temperatures in an inert environment, thermogravimetric analysis was carried out by using (Perkin Elmer, Pyres Diamond series TG/DTA, USA). The crystal structure, lattice parameters, and particle size of crystallite were studied using an X-ray diffractometer (JDX-3532, JEOL, Japan). JSM5910’s energy dispersive X-ray spectroscopy (EDX) was used to analyze the sample elemental analysis. Shimadzu IR Prestige-21 FT-IR spectroscopy was also used to examine the samples in order to investigate various functional groups.

### 2.3. Antimicrobial Activity

Two pathogenic bacteria, *Staphylococcus aureus* (ATCC 6538) and *Klebsiella pneumoniae* (ATCC 13883), as well as a fungal human pathogen, *Candida albicans*, were tested for Ag-NPs antimicrobial potential (Cl.l 4043). The Department of Microbiology at Quaid-i-Azam University Islamabad provided all of the strains. DMSO was used to disperse the Ag-NPs to a test concentration of 5 mg/mL. All of the samples antimicrobial activity was performed by using an agar as standard by applying the well diffusion method. Suspension of fresh cultures was made and their turbidity was adjusted to the MacFarland standard of 0.5 BaSO_4_. To develop the microbial lawn, microbial suspensions were evenly distributed on nutrient agar plates. Then, an 8 mm diameter well was drilled using a sterile cork borer. Each well received 100 µL of NP suspension in equal amounts. Under the positive control of nystatin (4400 USP units/mg) and cefixime-USP (20 µg) drug, while DMSO was used as the negative control. For 24 h, all of the agar plates were incubated at 37 °C. The diameter of the zone of inhibition was measured in millimeters. Each test was performed three times and the mean value was recorded.

### 2.4. Catalytic Activity

The degradation of Allura red and fast green dyes was used to perform the catalytic performance of prepared Ag-NPs. Each dye was dissolved in 100 mL of deionized water at a concentration of about 0.001 g and the absorption spectra were recorded using a UV-visible spectrophotometer. Then, 0.1 g of NaBH_4_ was added and the absorption spectra were recorded again. Then, using a UV-visible spectrophotometer to measure the degradation activity, 0.01 g of Ag-NPs were added to 100 mL solutions of each dye and exposed to visible light for a predetermined amount of time.

## 3. Results and Discussion

### 3.1. Photoluminescence Spectroscopy

**Figure 2:**
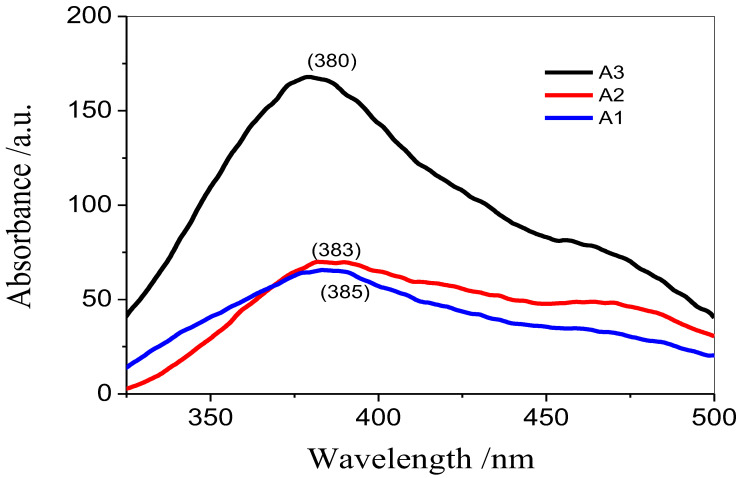
Photoluminescence emission spectra of silver nanoparticles prepared by using clove oil, cinnamon oil, and cardamom oil.

Photoluminescence spectroscopy in the range of 300 to 500 nm was used to analyze the effectiveness of clove oil, cinnamon oil, and cardamom oil in the synthesis of Ag-NPs. Surface plasmon resonance bands were used for the confirmation of the NP [25,26]. The mechanism for the formation of silver nanoparticles is depicted in Figure 2 and the change in the color of the solution from milky white to yellowish brown confirms the formation of Ag nanoparticles and reduction of Ag^+^ ions into Ag atoms. Surface plasmon resonance bands were used to observe the luminescence emitted by all of the Ag-NP samples that were manufactured by making use of essential oils. The increase in the size of nanoparticles is responsible for the shift in the plasmon emission maxima to a region with higher wavelengths [27]. In addition to its other components, clove oil has eugenol as its primary component, which accounts for 75–80 percent of the total content. The phenolic ring that is part of the structure of eugenol is responsible for the reducing character of the compound, which is accomplished by the donation of a proton (H^+^) [28]. Eugenol has an amphiphilic nature; however, it does not efficiently prevent clustering in the nanoparticles. This may be related to its log P value of 2.10 and topological polar surface area (TPSA) of 29.46 [29]. In comparison, cinnamaldehyde is the main component of cinnamon oil, which acts as a capping and reducing agent. It has a log P value of 2.48 and TPSA of 17.07, this explains its efficiency to prevent agglomeration of silver nanoparticles size control in the case of cinnamon [29]. The antimicrobial activity of cinnamon oil is also due to cinnamaldehyde [30]. The major compounds of cardamom essential oil are 1,8-cineole (log P 2.72, TPSA 9.23), α-terpineol (log P 2.60, TPSA 20.23), α-terpinyl acetate (log P 3.30, TPSA 26.30), d-limonene (log P 3.62, TPSA 0.00), sabinene (log P 3.10, TPSA 0.00), and borneol (log P 2.35, TPSA 20.23) [31]. These values of TPSA and log P were determined using the molinspiration online property calculator. Cardamom oil was anticipated to have less impact on the particle size and homogeneity of the final product as a result of the wide variety of antioxidants and capping agents it contains, which ranges from around 27 to 32 percent 1,8-cineole [32].

### 3.2. Dynamic Laser Light Scattering

The hydrodynamic radius of the synthesized nanoparticles was calculated by using a dynamic laser light scattering device (a Brookhaven Institute and Cor. 90 plus particle size analyzer) in addition to the polydispersity index (PDI). The size distribution of the Ag-NPs can be represented by their PDI value. When the PDI values are lower, this indicates that the particle size distribution is more uniform, meaning that all the chains are of the same length. In addition, low PDI values suggest homogeneity in the particle size distribution [33]. Table 1 shows that silver nanoparticles prepared by using clove oil, cinnamon oil, and cardamom oil have almost the same behavior, i.e., almost the same values of average effective diameter (80–92 nm) with low PDI (0.005).

### 3.3. X-ray Diffraction

Powdered XRD analysis was performed with the assistance of an X-ray diffractometer in order to investigate the crystallite structures of silver nanoparticles (JDX-3532, JEOL, and Japan). Clearly, the XRD patterns exhibit prominent peaks (2Ө) detected at 38.12°, 44.36°, 64.48°, and 77.5°, which corresponds to (111), (2 00), (22 0), and (311) Bragg reflections accordingly. These peaks can be found at each of these respective angles. In the (111), (200), (220), and (311) planes of Ag with a face-centered cubic crystal structure, all of the planes have a matching relationship with the 2 theta (degree) [33]. These peaks provided evidence that silver nanoparticles had been formed and that they are pure. It has been proved beyond a reasonable doubt that the XRD peaks for each of the samples were located at the same place as depicted in the literature, with the only difference in the intensity of their peak, this variation corresponds to the sample’s overall concentration. For the purpose of determining the crystallite size of each sample, the Scherer equation was employed. Where K is the Scherer constant with a value that ranges from 0.9 to 1, λ is the wavelength of the X-ray, β is the full width at half maximum, θ is the Braggs angle, and D is the average crystallite size [34].

$$D=\frac{K\lambda }{\beta \mathrm{COS}\theta }$$

**Figure 3:**
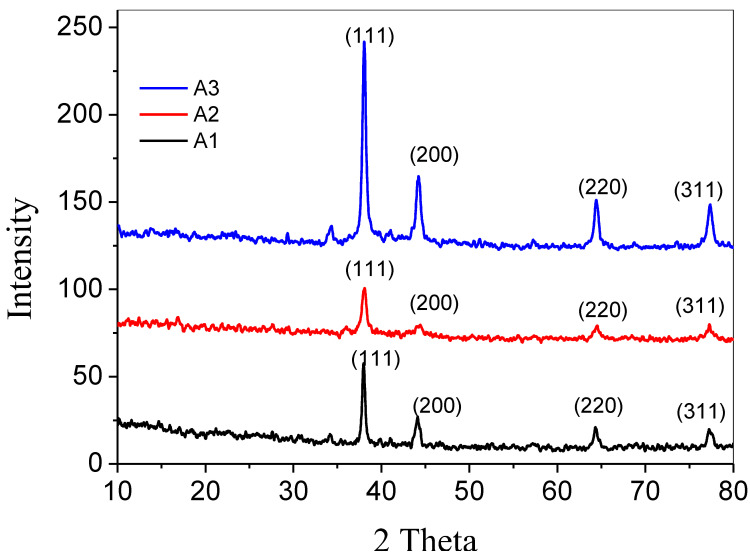
XRD patterns of silver nanoparticles synthesized by using clove oil, cinnamon oil, and cardamom oil.

In XRD, the average crystallite size calculated was smaller than the hydrodynamic radius calculated from DLS [35]. The dislocation density (d), which corresponds to the number of defects in the sample and is determined by the length of dislocation lines per unit volume of the crystal, was calculated by using the Scherer equation. The value of d can be found by multiplying the length of dislocation lines with the volume of the crystal.

$$\delta =\frac{1}{{D\;}^{2}}$$

where D is the crystallite size and δ is the dislocation density [36]. Below, Table 2 shows the average values of crystallite sizes and the dislocation densities of the three samples of Ag-NPs. The average crystallite size of silver nanoparticles synthesized by using clove oil was found to be greater than those synthesized by using cinnamon oil with smaller crystal defects and dislocation density. The data acquired from Figure 3 were analyzed by using X’Pert HighScore Plus software.

### 3.4. FT-IR Spectroscopy

**Figure 4:**
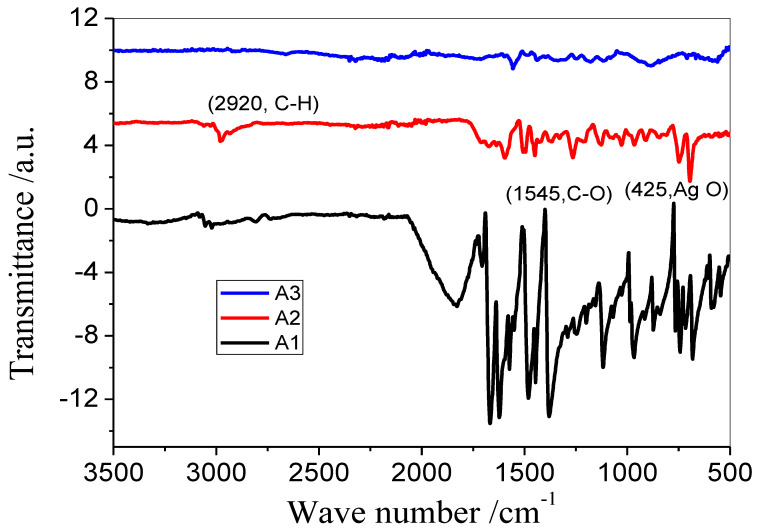
FTIR analysis of silver nanoparticles prepared by using clove oil, cinnamon oil, and cardamom oil.

In order to determine the variety of distinct functional groups present or not in the samples, FT-IR spectra were analyzed. Clearly, the FTIR spectra (which were acquired using a Perkin-Elmer FTIR spectrometer with a spectral range of 500–4000 cm^−1^) displayed a variety of peaks in both the fingerprint region and the functional group region. The link between Ag and O is corresponding to the peak in the FTIR spectrum at 425 nm. The primary peaks found (Figure 4) exhibit functional groups with the formulas C-O, C-H, C=O, and O-H. These peaks are incorporated into the Ag-NPs in the form of a variety of reducing agents present in the selected oils and ether used for washing.

Major peaks identified corresponded to C-H, C=O, O-H, and C-O groups. It is known that the valence electron configuration of the Ag ion is 4d^10^, 5s^0^. So, there are empty s, p, and d orbitals that can form coordination bonds by accepting a lone pair of electrons or can be absorbed by electrostatic attractions. On the surface of clove microemulsion, silver ions undergo oxidation. Thus, silver ions become reduced by phenols and other reducing agents present in cinnamon oil and other oils. In the reduced silver there still exist empty p and d orbitals. The hydroxol, carboxyl, and ketone groups found in the oils can act as electron pairs. Thus, reduced Ag was adsorbed on the microemulsion surface and wrapped in macromolecules to develop Ag nanospheres, which were then separated into aromatic and alyl groups [37].

### 3.5. TGA Analysis

**Figure 5:**
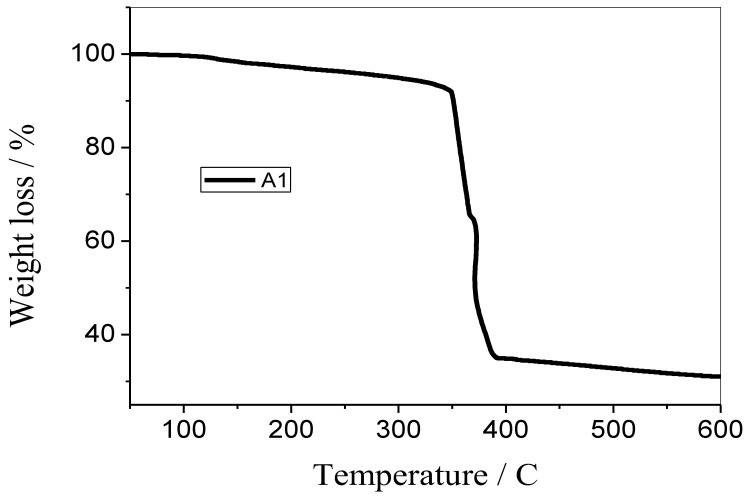
TGA analysis of silver nanoparticles synthesized by using clove oil.

The samples’ weight loss was measured using a thermogravimetric analyzer (Perkin Elmer’s Pyres Diamond series TG/DTA, which was manufactured in the United States) in order to determine the samples’ thermal stability and the proportion of their volatile components. Since clove oil, cinnamon oil, and cardamom oil have nearly close values for melting and boiling points that are above 200 °C, that is why only the A1 sample system was subjected to heating at a consistent pace in an environment of nitrogen. Figure 5 exhibited the thermal stability and percentage of volatile components in sample A1. It was noted that 70% of the sample degraded at 600 °C, thus demonstrating the extremely high thermal stability of the Ag-NPs prepared by using clove oil. At about 200 °C, the volatile components begin to disintegrate. The selected sample thus exhibits extremely high resistivity towards weight loss [38].

### 3.6. EDX Analysis

**Figure 6:**
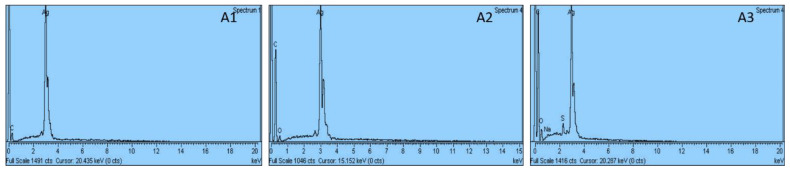
EDX images of Ag-NPs synthesized by using clove oil, cinnamon oil, and cardamom oil.

The EDS analysis confirmed the presence of Ag atoms in the synthesized samples (Figure 6). As can be seen, the spectrum’s most prominent peak showed that the sample contains Ag atoms along with a small amount of other capping agents and reducing agents that remain adhered to the sample after washing that contain sulphur, oxygen, carbon, and sodium atoms. These peaks are due to the existence of a trace amount of the above elements that were not removed throughout the purification process [39].

### 3.7. Transmission Electron Microscopy

**Figure 7:**
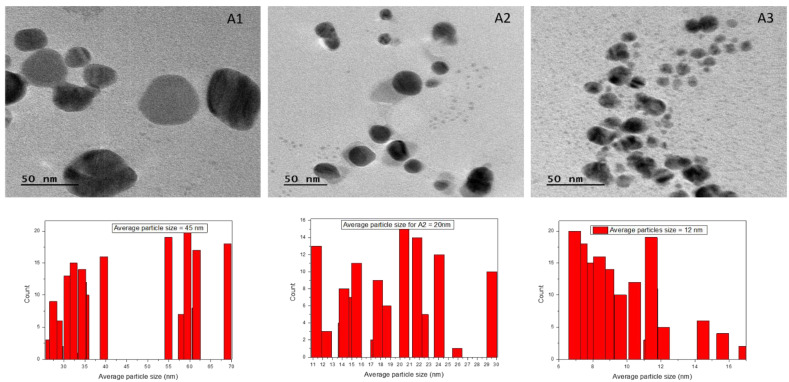
TEM images of Ag-NPs synthesized by using clove oil, cinnamon oil, and cardamom oil.

TEM examination was performed on the samples so that the exact particle size could be studied. The TEM micrographs (Figure 7) revealed that the majority of the nanoparticles have a spherical shape, while there are also some nanoparticles with irregular flake shapes. The examination of these TEM images revealed that the average particle size grows when there is a reduction in the proportion of clove extract to AgNO_3_ in the mixture [40]. The average particle size for the A1 system calculated from Image J software was found to be 45 nm, for A2 was 20 nm, and for A3 was 12 nm.

### 3.8. Antimicrobial Activity

**Figure 8:**
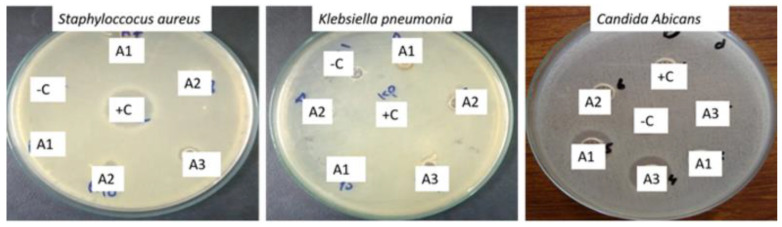
Antimicrobial activities of silver nanoparticles synthesized by using clove oil, cinnamon oil, and cardamom oil.

For antimicrobial studies, the prepared samples of silver nanoparticles activity were studied against two bacterial species, *Staphyloccocus aureus* (ATCC 6538) and *Klebsiella pneumoniae* (ATCC 13883), and a human fungal pathogen, i.e., *Candida albicans* (Cl.l 4043). Clove oil also harvests a vital antimicrobial effect on silver nanoparticles. The enhanced antibacterial activity of silver nanoparticles finds its applications in wound healing [41]. Cefixime-USP (20 µg/well-Sigma Aldrich, zone of inhibition 23 ± 4 mm) and Nystatin (zone of inhibition 1.04 ± 20.46) were applied as the positive control, whereas DMSO was the negative control. All three systems of silver nanoparticles had shown very good antibacterial and antifungal activities with a clear zone of inhibition with a minimum value of zone of inhibition of 6 mm to a maximum value of 24 mm, as shown in Figure 8 and Table 3. Thus, the A1 sample had shown the strongest antimicrobial activities.

### 3.9. Photocatalytic Activity

**Figure 9:**
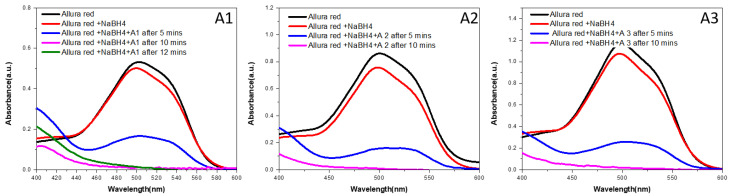
Dye degradation curves of A1, A2, and A3 for Allura red.

**Figure 10:**
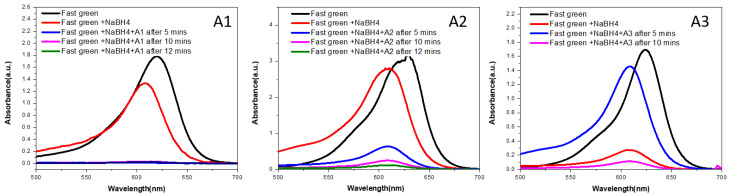
Dye degradation curves of A1, A2, and A3 for fast green.

The photocatalytic behavior of the selected compounds against Allura red and fast green dye are depicted in Figure 9 and Figure 10, respectively. Both of these dyes are utilized in the food coloring industry, despite the fact that they are unhygienic [42]. It is possible to observe the powerful activity of prepared samples against these dyes by observing the destruction of the dyes when they are irradiated with UV-visible light. Silver nanoparticles basically act as electron trapping sites as a result increased visible light absorption and band gap narrowing occur. The photo-induced electrons produce reactive oxygen species having strong oxidation abilities. The reactive oxygen species are basically responsible for the degradation of the organic dye [43]. The data shown in Figure 9 and Figure 10 make it abundantly evident that the produced materials demonstrated extremely strong photocatalytic activity by destroying Allura red dye in less than ten minutes, in contrast to fast green dye, which was totally degraded in approximately fifteen minutes. For the purpose of comparison, the dye degradation spectra of silver nanoparticles that were manufactured by using clove oil, cinnamon oil, and cardamom oil make it abundantly clear that the sample containing clove oil catalytic activity is the most potent compared to samples containing other oils for both Allura red and fast green dye.

### 3.10. Comparison with Literature

In this section a comparison is made between the present work and work already reported in the literature with the specification of materials used and particle size obtained i.e., only green synthesis of Ag-NPs by using oils is compared. In this context, V.S. Christopher in 2021 published an article that reported turmeric oil mediated green synthesis of silver nanoparticles and studied their antioxidant activity [44]. Similarly, R.D. Rivera-Rangel in 2018 carried out green synthesis of Ag-NPs by using geranium aqueous leaf extract along with castor oil and surfactant. The amount and concentration of a metallic precursor and geranium leaf extract (GLE) in the systems used allowed synthesizing Ag-NPs with sizes ranging from 25 to 150 nm [45]. A.A. Alfuraydi et al. in 2019 used sesame oil for the synthesis of silver nanoparticles and studied their potential anti-cancerous and antimicrobial activity and 6.6–14.8 nm particle size was calculated from the TEM analysis [46]. H. Veisi et al. in 2019 prepared silver nanoparticles based on the oil-water interface method with the essential oil of orange peel. Ag/EOs orange NPs indicated high catalytic activity for the A3 coupling reaction and calculated particle size to be around 2.76 nm [1]. M.V.D.O.B. Maciel et al. in 2019 used clove essential oil for the synthesis of Ag-NPs and studied their antimicrobial activities and calculated their average size ranging from 27 to 94 nm [14]. S.W. Ahmed et al. in 2018 used olive oil for the synthesis of Ag-NPs and studied its chemo sensing of nitrofurazone and also its antimicrobial and biofilm inhibition activity [47]. V. Vilas et al. in 2014 used the essential oil of *Myristica fragrans* enriched in terpenes and phenyl propenes for the synthesis of silver nanoparticles and studied its antioxidant, chemo catalytic and antimicrobial activities, and the particle size calculated from TEM analysis was found to be 12–26 nm [48]. B.S. Vasile et al. in 2020 prepared wound dressings coated with Ag-NPs and essential oils (mandarin, niaouli, and clove) to cure wound infections. The average particle size calculated was 69 nm [49]. J. González-Rivera et al. in 2017 carried out microwave-assisted synthesis of Ag-NPs by using rosemary essential oil as a reducing agent and calculated its antioxidant and antimicrobial activity [50]. M.V.D.O.B. Maciel et al. in 2020 synthesized silver nanoparticles by using oregano (*Origanum vulgare*), thyme (*Thymus vulgaris*), clove (*Syzygium aromaticum* L.), rosemary (*Rosmarinus officinalis* L.), and *Poiretia latifolia*. They studied their antimicrobial activity and suggested their use in food packaging and calculated the average value of particle size to be 18.6–22.4 nm [51]. L. Motelica et al. in 2021 synthesized antibacterial biodegradable films by using silver nanoparticles and lemon grass essential oil as an innovative packaging for cheese. The particle size of silver nanoparticles was calculated to be 5–25 nm (see Table 4) [52].

## 4. Conclusions

Clove oil proved itself to be a good capping and reducing agent but there is the problem of agglomeration (large particle size, log P value of 2.10, TPSA of 29.46) as compared to cinnamon oil with less chances of agglomeration (comparably small particle size, log P value of 2.48 and TPSA of 17.07). Cardamom oil (1,8-cineole, log P 2.72, TPSA 9.23), due to the diversity of antioxidants and capping agents with 1,8-cineole ranging between 27–32%, approximately, was expected to have a lesser effect on particle size and homogeneity and thus acted as a size reducer but did not prevent agglomeration in the samples. The average crystallite sizes calculated from XRD (16–23 nm) were found to be smaller than the hydrodynamic radius calculated from DLS (80–92 nm). DLS measures particle size in dispersion, which includes material attached to the particle. TEM analysis reveals the actual size after drying the sample. The TEM images revealed the average particle size of A1 was 45 nm (spherical), while the average particle size of A2 was calculated to be 20 nm (elongated), and for A3 was 12 nm (elongated). The UV-spectrums for dye degradation demonstrated very strong photocatalytic activity in all three samples. All the prepared samples of silver nanoparticles had shown very good antibacterial activity, with a distinct zone of inhibition, 6–24 nm. An increased surface area results in a smaller particle size, which in turn leads to a higher level of antimicrobial activity. The results obtained strongly suggest the potential application of the synthesized nanoparticles in wound healing and wastewater treatment (Table 5).

## Figures and Tables

**Table 1:** Effective diameter and polydispersity index of prepared samples of Ag-NPs.

Sr. No.	Sample Name	Diameter (nm)	Polydispersity Index
1.	A1	92	0.0057
2.	A2	87.3	0.005
3.	A3	80.1	0.005

**Table 2:** Crystallite size and dislocation densities of prepared samples.

Name of Sample	Average Crystallite Size (nm)	Dislocation Density (δ)
A1	23.2	1.8 × 10^−3^
A2	19.68	2.58 × 10^−3^
A3	16.18	3.8 × 10^−3^

**Table 3:** Zone of inhibition (mm) for antimicrobial studies of essential oils-based synthesis of silver nanoparticles.

Name of Species	Zone of Inhibition (mm)		
	A1	A2	A3
*Staphyloccocus aureus*	15	12	18
*Klebsiella pneumonia*	23	21	24
*Candida albicans*	16	6	10

**Table 4:** Synthesis of silver nanoparticles using essential oils and their potential applications.

S. NO.	Synthesis Method	Composition	Average Particle Size (NM)	Application	Ref.
1	Green synthesis	AgN0_3_ + Turmeric oil	-------	Biomedical and antioxidant activities	[44]
2	Green synthesis	Silver Stearate + Castor oil + Birj surfactant	25–150	-------	[45]
3	Green synthesis	AgNO_3_ + Sesame oil	6.6–14.8	Anticancer and antimicrobial	[46]
4	Green synthesis	AgNO_3_ + Orange peel oil extract	2.76	Nano catalysis	[1]
5	Green synthesis	AgNO_3_ + Clove oil _+_ NaOH	27–94	Antibacterial activity	[14]
6	Green synthesis	AgNO_3_ + Olive oil	------	Antimicrobial and antibiofilm	[47]
7	Green synthesis	AgNO_3_ + leaves of *M. fragrans + NaBH_4_*	-------	Antioxidant and antimicrobial	[48]
8	γ-radiation method	AgNO_3_ + NaOH + Glucose	69	Wound healing	[49]
9	Green synthesis	Silver Acetate + Rosemary essential oil + Microwave	7–18	Compounds synthesis	[50]
10	Green synthesis	AgNO_3_ + essential oils + methanol	------	Antimicrobial and food preservation	[51]
11	Green Synthesis	AgNO_3_ + sodium citrate + Lemon grass essential oil	-------	Antimicrobial films for Cheese preservation	[52]
12.	Green synthesis	Ag NO_3_ + Clove oil + Cinnamon oil + Cardamom oil	8–100	Photocatalytic and antimicrobial application	This work

**Table 5:** Comparison of particle size from DLS, XRD, and TEM results of silver nanoparticles.

Sr. No.	Sample Code	Average Effective Diameter from DLS Analysis	Average Value of Crystallite Size from XRD Analysis (nm)	Average Value of Particles Size from TEM Analysis (nm)
1.	A1	92	23.2	45
2.	A2	87.3	19.68	20
3.	A3	80.1	16.18	12

## Data Availability

The data that support the findings of this study are available from the corresponding author upon reasonable request.
